# A Narrative Review on the Relationship between Female Reproductive Factors and Longevity

**DOI:** 10.1093/geroni/igaa057.3269

**Published:** 2020-12-16

**Authors:** Christy Costanian, Raymond Farah, Sola Bahous, Abla Sibai

**Affiliations:** 1 Lebanese American University, Beirut, Lebanon; 2 Lebanese University, Beirut, Lebanon; 3 American University of Beirut, Beirut, Lebanon

## Abstract

This review presents findings on the role of female reproductive factors on longevity.A comprehensive systematic literature search was conducted using four electronic databases: OVID Medline, Web of Science, PubMed and Google Scholar from inception until May 2020 and restricted to English language articles that tackle the relationship between reproductive factors and longevity in its various definitions. Our search yielded a total of 306 articles. After screening based on the eligibility criteria,37 articles were included for review. The majority of studies were prospective and conducted in Western populations. The most consistent findings were between parity and increased longevity. The role of ages at menarche and menopause, premature menopause, as well as reproductive lifespan on longevity were not conclusive. Whether gender of offspring is related to maternal longevity is yet to be fully elucidated.Variations in findings are in the majority due to differentials in the definition of longevity as an outcome. Further longitudinal studies based in developing countries are needed to examine reproductive factors related to longevity.

